# Boron deficiency inhibits root cell elongation via an ethylene/auxin/ROS-dependent pathway in *Arabidopsis* seedlings

**DOI:** 10.1093/jxb/erv186

**Published:** 2015-04-28

**Authors:** Juan J. Camacho-Cristóbal, Esperanza M. Martín-Rejano, M. Begoña Herrera-Rodríguez, M. Teresa Navarro-Gochicoa, Jesús Rexach, Agustín González-Fontes

**Affiliations:** Departamento de Fisiología, Anatomía y Biología Celular, Universidad Pablo de Olavide, E-41013, Sevilla, Spain

**Keywords:** *Arabidopsis*, auxin, boron, ethylene, reactive oxygen species, root elongation.

## Abstract

One of the earliest symptoms of boron (B) deficiency is the inhibition of root elongation which can reasonably be attributed to the damaging effects of B deprivation on cell wall integrity. It is shown here that exposure of wild-type *Arabidopsis thaliana* seedlings to B deficiency for 4h led to a drastic inhibition of root cell length in the transition between the elongation and differentiation zones. To investigate the possible mediation of ethylene, auxin, and reactive oxygen species (ROS) in the effect of B deficiency on root cell elongation, B deficiency was applied together with aminoethoxyvinylglycine (AVG, a chemical inhibitor of ethylene biosynthesis), silver ions (Ag^+^, an antagonist of ethylene perception), α-(phenylethyl-2‐oxo)‐indoleacetic acid (PEO-IAA, a synthetic antagonist of TIR1 receptor function), and diphenylene iodonium (DPI, an inhibitor of ROS production). Interestingly, all these chemicals partially or fully restored cell elongation in B-deficient roots. To further explore the possible role of ethylene and auxin in the inhibition of root cell elongation under B deficiency, a genetic approach was performed by using *Arabidopsis* mutants defective in the ethylene (*ein2‐1*) or auxin (*eir1-4* and *aux1-22*) response. Root cell elongation in these mutants was less sensitive to B-deficient treatment than that in wild-type plants. Altogether, these results demonstrated that a signalling pathway involving ethylene, auxin, and ROS participates in the reduction of root cell elongation when *Arabidopsis* seedlings are subjected to B deficiency. A similar signalling process has been described to reduce root elongation rapidly under various types of cell wall stress which supports the idea that this signalling pathway is triggered by the impaired cell wall integrity caused by B deficiency.

## Highlight

The rapid inhibition of root cell elongation in response to boron deficiency is mediated by an ethylene/auxin/ROS dependent pathway

## Introduction

Boron (B) is an essential element for plant development whose limitation in soil and irrigation water influences the yield and quality of crops in many regions of the world (Shorrocks, 1997). Thus, B deficiency has been reported to reduce the yield of cotton, rice, maize, wheat, and soybean crops (Ahmad *et al.*, 2012). Insufficient B availability affects several physiological and metabolic processes in plants such as cell wall and plasma membrane structure and function, phenolics and nitrogen metabolisms, secondary metabolism and oxidative stress, gene expression, shoot and root growth (Cakmak and Römheld, 1997; Brown *et al.*, 2002; Goldbach and Wimmer, 2007; Camacho-Cristóbal *et al.*, 2008b, 2011; Herrera-Rodríguez *et al.*, 2010; González-Fontes *et al.*, 2013), among others.

Plants take up B as boric acid by three mechanisms: (i) passive transport across the plasma membrane by simple diffusion, (ii) facilitated transport carried out by NIP (nodulin intrinsic protein) channels, and (iii) energy-dependent high-affinity transport, mediated via BOR transporters (Brown *et al.*, 2002; Goldbach and Wimmer, 2007; Takano *et al.*, 2008). B, both as boric acid and borate, can form complexes with a wide variety of biological compounds containing two hydroxyl groups in *cis* configuration (Bolaños *et al.*, 2004; Goldbach and Wimmer, 2007), and this capacity has been proposed as the key of any B function in plants (Bolaños *et al.*, 2004). Thus, the main known function of B in plants is the establishment of borate esters with apiose residues of two rhamnogalacturonan II (RGII) molecules (Kobayashi *et al.*, 1996). These RGII-borate dimers cross-link pectins in the cell wall and form a three-dimensional pectic network which contributes to the mechanical properties of cell wall structure (Ryden *et al.*, 2003; O’Neill *et al.*, 2004). Interestingly, in a recent work it has been shown that B bridging of RGII occurs before polysaccharide secretion into the apoplast (Chormova *et al.*, 2014).

Due to the structural role that B plays in the cell wall, it cannot be ruled out that many of the effects associated with B deficiency could be the consequence of a disturbed cell wall structure. For instance, the most rapid response to B deficiency in vascular plants is the inhibition of root elongation in both the main and lateral roots (Dell and Huang, 1997). Total root elongation depends on two processes: cell division (mostly in the root meristematic region) and enlargement (in the elongation zone). Several physiological studies suggest that B deficiency affects cell elongation rather than cell division in the growing tissues of plants (Dell and Huang, 1997; Martín-Rejano *et al.*, 2011). This inhibition in cell elongation can reasonably be attributed to the adverse effects of B deprivation on the physical stability of the cell wall which is essential for the cell elongation process (De Cnodder *et al.*, 2005). Nevertheless, our understanding of how and why the perturbed cell wall structure caused by B deficiency leads to an inhibition of cell elongation is still limited.

It is well known that rapidly expanding cells (i.e. during the growth of etiolated hypocotyls and primary root tips) respond dramatically to a perturbation of cell wall integrity. Previous studies have reported that this response is not only an inevitable mechanical consequence of the loss of cell wall integrity, but rather it is mediated by a cell wall integrity-sensing mechanism and dedicated signalling pathway (Refrégier *et al.*, 2004; Tsang *et al.*, 2011). Recently, in a fine work using isoxaben—a cellulose synthase inhibitor that causes perturbation in the cell wall—it was described that cell wall integrity controls root elongation via an ACC-dependent pathway that requires the downstream participation of auxin signalling as well as the production of reactive oxygen species (ROS) (Tsang *et al.*, 2011). Interestingly, there is growing evidence suggesting the possible link between B deficiency and ethylene, auxin, and/or ROS signalling during the inhibition of *Arabidopsis* root growth (Martín-Rejano *et al.*, 2011; Oiwa *et al.*, 2013). Therefore, taking into account all this evidence, the possible occurrence of a dedicated signalling pathway to control root cell elongation under B deficiency was investigated. For this purpose, the effect of short-term B deficiency on the length of root cells in the transition between the elongation and differentiation zones was studied in detail. Our results indicate that B deficiency rapidly reduces root cell elongation in *Arabidopsis*; by using different experimental approaches it was concluded that this response is driven by a signalling pathway with the participation of ethylene, auxin, and ROS production.

## Materials and methods

### Plant material and growth conditions

The following mutants and transgenic lines were obtained from the European *Arabidopsis* Stock Centre (http://arabidopsis.info/): *ein2-1* (N3071), *aux1-22* (N9585), and Theo-At-ACS11-GUS/GFP (N31387). The *eir1-4* (*pin2*) mutant and the IAA2::GUS reporter lines were kindly provided by Dr C Luschnig (University of Natural Resources and Life Sciences, Vienna, Austria) and Dr P Doumas (INRA, Montpellier, France), respectively.

Seeds of wild-type (ecotype Col-0) and the different *Arabidopsis* lines described above were surface-sterilized with 75% (v/v) ethanol for 5min, then 2% (w/v) hypochlorite solution for 5min and, finally, washed six times with sterile water. Sterile seeds were sown on square (12×12cm) plates containing 40ml of sterile culture medium and sealed with Parafilm. The culture medium contained 1mM Ca(NO_3_)_2_, 1mM KNO_3_, 0.5mM MgSO_4_, 0.75mM KH_2_PO_4_, 12.5 μM NaCl, 12.5 μM FeNa-EDTA, 2.5 μM MnCl_2_, 0.5 μM ZnSO_4_, 0.25 μM CuSO_4_, 0.125 μM Na_2_MoO_4_, 0.05 μM CoCl_2_, 10 μM H_3_BO_3_, 2mM MES, and 0.5 % (w/v) sucrose, adjusted to pH 5.7 with KOH and solidified with 1% (w/v) Phytagel (P8169, Sigma-Aldrich). After incubation at 4 °C for 5 d in darkness to promote and synchronize germination, the plates were transferred to a growth chamber in a vertical orientation with a light/dark regime of 16/8h, 25/22 °C, 75/75% relative humidity, and a light intensity of 120–150 μmol·m^–2^·s^–1^ of photosynthetically active radiation. Seedlings were grown in these conditions for 5 d and then used for further analysis.

### Root treatments

At least 20 5-d-old seedlings were carefully transferred to new plates containing solidified B-deficient medium (no B added) or control medium (10 μM B) typically for 4h. When indicated, the following reagents were added to the media before solidification: aminoethoxyvinylglycine (AVG), silver thiosulphate [Ag^+^ (a 20mM stock was freshly prepared by mixing 1vol. of 100mM silver nitrate with 4 vols of 100mM sodium thiosulphate)], 1-aminocyclopropane-1-carboxylic acid (ACC), ethephon (from 5mM stock mixed with an equal volume of 15mM HEPES/KOH, pH 6.5), α-(phenylethyl-2-oxo)-IAA (PEO-IAA, a kind gift of Dr Ken-ichiro Hayashi), or diphenylene iodonium (DPI). All chemicals were from Sigma-Aldrich unless noted otherwise.

### Root elongation and LEH measurements

After the 4h of treatments, images of the root system were recorded directly from plants growing in Petri dishes using a desktop scanner (resolution: 450 dpi). Images corresponding to different growth times were analysed using Optimas software version 6.1 (Media Cybernetics, MD, USA). The length of the primary root was determined manually. Data were exported to an Excel work-sheet for final processing. Primary root elongation was calculated by subtracting the primary root length at time 0 from the primary root length at the indicated time.

For the measurement of the length of the first epidermal cell with a visible root hair bulge (LEH; Le *et al.*, 2001), primary roots were analysed with a Leica S8APO Stereozoom microscope and the images captured with a digital camera (Leica EC3) driven by Analysis software (LAS EZ, Switzerland). LEH was measured as the distance from the first visible root-hair bulge to the next more differentiated root hair in the same trichoblast cell file (Tsang *et al.*, 2011) by using Optimas software version 6.1. On each root, one to three cells could be measured with confidence, resulting in 30–50 measurements per treatment. Data were exported to an Excel work-sheet for final processing.

Values are given as the mean ±SD of at least 20 separate plants. Each result is representative of at least three independent experiments. All results were statistically analysed using the Student’s *t* test.

### GUS staining, reactive oxygen species (ROS) localization

For histochemical analysis of GUS reporter enzyme activity, *Arabidopsis* seedlings were incubated at 37 °C in a GUS reaction buffer containing 2mM 5-bromo-4-chloro-3-indolyl-β-d-glucuronide in 100mM sodium phosphate (pH 7.0). GUS staining patterns were analysed on a Leica S8APO Stereozoom microscope equipped with a digital camera (Leica EC3) driven by Analysis software (LAS EZ, Switzerland).

The pattern of ROS accumulation in root tips was detected using dihydroethidium (DHE) (Oiwa *et al.*, 2013). For this purpose, *Arabidopsis* seedlings were incubated with 10 μM DHE for 30min in the dark. After that, roots were observed for ethidium fluorescence with a fluorescent microscope (Zeiss Axioskop) equipped with a 510–560nm excitation filter and a 590nm barrier filter.

For each plant line and for each treatment, at least 10 plants were analysed in two independent experiments. Representative plant images were chosen for each B treatment.

### NADPH oxidase activity in roots

Enzyme extraction and activity was performed according to a modified method of Ozgur *et al.* (2014). Briefly, root samples (0.1g) were ground in 500 μl of extraction buffer containing 50mM TRIS–HCl (pH 7.5), 0.1mM EDTA, 0.1% (w/v) Triton-X100, 1mM PMSF, and 1% (w/v) PVP, and the extracts were centrifuged at 14000× *g* for 15min at 4 °C. Total soluble protein contents of the enzyme extracts were determined according to Bradford (1976) using BSA as a standard. NADPH oxidase (EC 1.6.3.1) activity was carried out by incubating the enzyme extracts (100 μl) in a substrate solution containing 50mM TRIS–HCl buffer (pH 7.5), 0.5mM XTT, and 100 μM NADPH. XTT reduction was followed at 470nm. The corrections for background reduction were determined in the presence of 10mM ZnCl_2_. Activity was calculated using the extinction coefficient 2.16×10^4^ M^–1^ cm^–1^. One unit of activity was defined as 1 μmol XTT reduced min^−1^. The specific enzyme activity was expressed as U mg^–1^ protein.

### RNA isolation, cDNA synthesis, and quantitative RT-PCR analyses

Total RNA extraction, cDNA synthesis, and quantitative RT-PCR (qRT-PCR) reactions were carried out following Beato *et al.* (2010).

The amplicon of *TON1A* gene (GenBank accession AF280058.1) (forward primer: TGTGAGGGATGGAACAAATG; reverse primer: AACGCAGTTGCAAATAAAGGA) was used as an internal control to normalize all data. The following gene-specific primers were used for quantitative RT-PCR analysis: *ACS11* (Gene ID: 826317) (forward primer GCCGAGCATTCTTTATGGAC, reverse primer CCATAGCAACCTCCATCGTT), *AUX1* (Gene ID: 818390) (forward primer AGACGCACTTCTCGACCACTCCA, reverse primer GCATCCCAATCACTTTCTCCCACA), and *PIN2* (Gene ID: 835813) (forward primer CGATACGACCCAAAGGTGAT, reverse primer CACCTAAGCCTGACCTGGAA). Efficiency of qRT-PCR reactions was higher than 95%.

Quantitative RT-PCR reactions were carried out with cDNA synthesized from five pools of 14 roots harvested randomly. The data shown are mean values ±SD. Results were statistically analysed using Student’s *t* test.

## Results

### Effect of B deficiency on root elongation

**Fig. 1.**
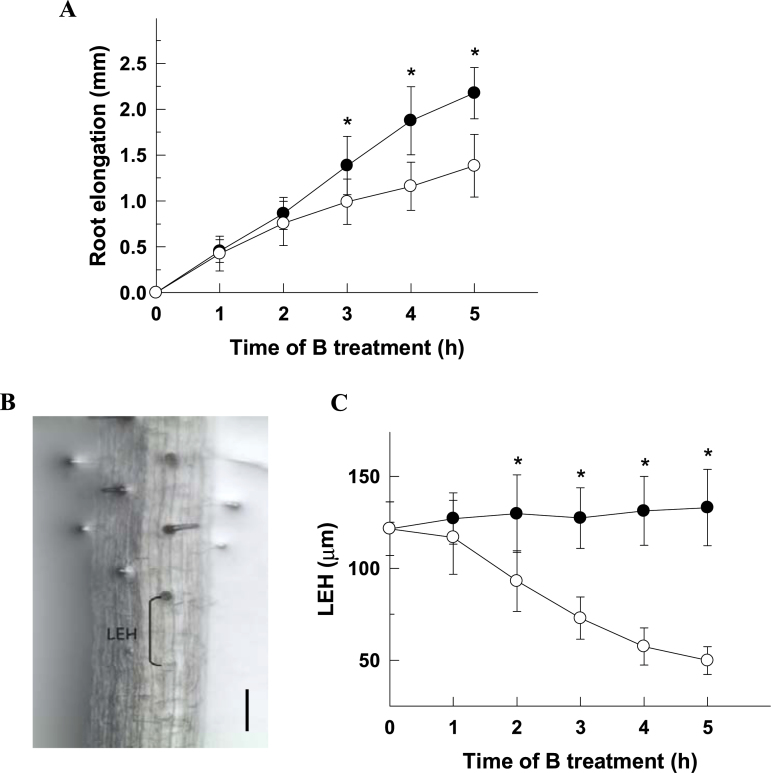
Effect of B deficiency on root elongation. (A) Time-course of root elongation in control (filled circles) and B-deficient (open circles) roots during 5h of treatments. (B) LEH was measured as the distance from the first visible root hair bulge to the next more differentiated root hair in the same trichoblast cell file. Scale bar=0.1mm. (C) Time-course of LEH in control (filled circles) and B-deficient (open circles) roots during 5h of treatments. The results are given as means ±SD (*n*=20 separate plants). Asterisks indicate statistically significant differences between B treatments at the referred times according to Student’s *t* test (*P* <0.001).

*Arabidopsis* seedlings were grown in 10 μM B for 5 d and then transferred to a B-deficient medium (0 μM B) or a control medium (10 μM B) for 5h. A temporal analysis of both primary root and root cell elongation was carried out every 1h (Fig. 1A, C). B deficiency significantly reduced primary root elongation when compared with the control treatment from 3h onwards (Fig. 1A). After 5h of B treatment, the elongation of the primary root was about 40% lower in B-deficient plants (1.38±0.34mm) than in control ones (2.18±0.28mm). The growth rate of *Arabidopsis* roots is mainly controlled by the length reached by individual cells in the elongation zone (Le *et al.*, 2001). Therefore, to study in detail the effect of short-term B deficiency on root elongation, the length of root cells in the transition between the elongation and differentiation zones was measured. To simplify this measurement, the length of the first epidermal cell with a visible root hair bulge (LEH; Fig. 1B; Le *et al.*, 2001; De Cnodder *et al.*, 2005) was used, a parameter that reflects rapid effects on elongation much more sensitively than macroscopic root length measurements (Tsang *et al.*, 2011). B deficiency significantly reduced the LEH to about 30, 45, 55, and 60% of the control within 2, 3, 4, and 5h, respectively (Fig. 1C). For the following experiments, 4-h treatments were chosen which led to a robust response.

### Involvement of ethylene in the rapid inhibition of LEH under B deficiency

**Fig. 2.**
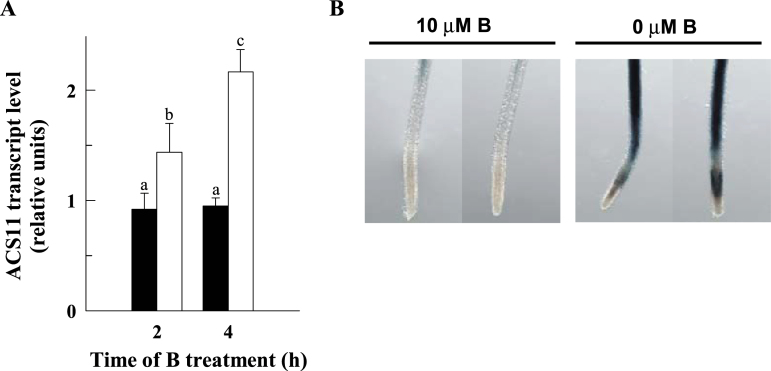
Boron deficiency induces the expression of the *ACS11* gene. (A) Quantitative RT-PCR analysis of *ACS11* transcript levels in control (filled bars) and B-deficient (open bars) roots after 2h and 4h of B treatments. The results are given as means ±SD (*n*=5 separate pools). Different letters are used to indicate means that differ significantly (*P* <0.001) according to Student’s *t* test. (B) GUS expression in roots of proACS11::GUS seedlings grown in a B-deficient (0 μM B) or control (10 μM B) medium for 4h. Images are representative individuals of two independent experiments with at least 10 seedlings examined for each experiment. (This figure is available in colour at *JXB* online.)

**Fig. 3.**
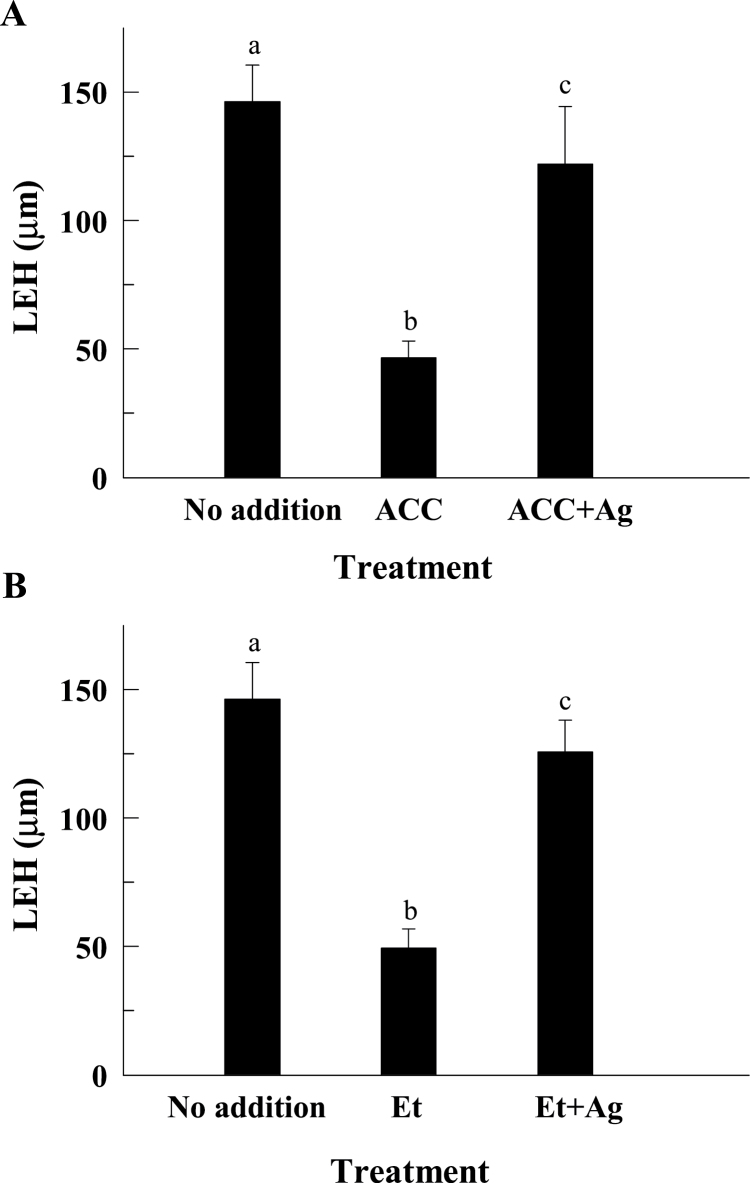
Effect of ACC and ethephon on root cell elongation. (A) The LEH parameter was measured in roots after 4h of treatments with 10 μM ACC or 10 μM ACC plus 10 μM Ag^+^. (B) The LEH parameter was measured in roots after 4h of treatments with 200 μM ethephon (Et) or 200 μM Et plus 10 μM Ag^+^. The results are given as means ±SD (*n*=20 separate plants). Different letters are used to indicate means that differ significantly (*P* <0.001) according to Student’s *t* test.

**Fig. 4.**
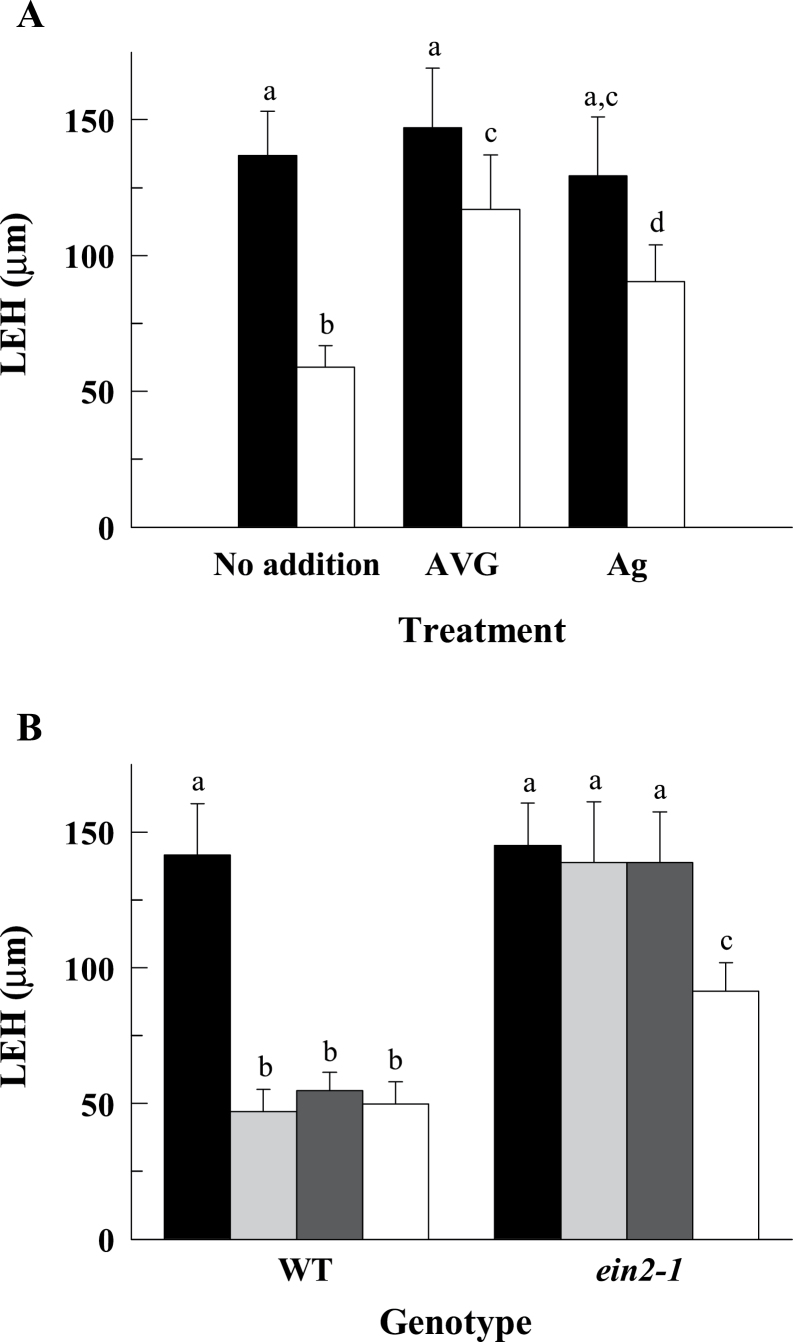
The inhibition of ethylene biosynthesis or action partially mitigated the repression of root cell elongation under B deficiency. (A) The LEH parameter was measured in control (black bars) and B-deficient (white bars) roots after 4h of B treatments in the presence (or not) of 10 μM AVG or 10 μM Ag^+^. (B) The LEH parameter was measured in control (black bars), control plus 10 μM ACC (light grey bars), control plus 200 μM ethephon (dark grey bars), and B-deficient (white bars) roots of wild-type and *ein2-1* mutant plants after 4h of B treatments. The results are given as means ±SD (*n*=20 separate plants). Different letters are used to indicate means that differ significantly (*P* <0.001) according to Student’s *t* test.

It is well known that ethylene affects root growth by inhibiting the rapid expansion of cells leaving the root meristem (Le *et al.*, 2001; Swarup *et al.*, 2007). Therefore, it was tested whether ACC synthase (ACS) expression, a key enzyme in the biosynthesis of ethylene, was induced in response to B deficiency. For this purpose, expression levels of several ACS isoforms were analysed by quantitative RT-PCR and histochemically by using pACS::GUS lines (Tsuchisaka and Theologis, 2004). Root *ACS2*, -*4*, -*5*, -*6*, -*8*, and -*9* expression was not significantly affected by B deficiency (see Supplementary Fig. S1 at *JXB* online); by contrast, root *ACS11* was strongly induced within 4h of B deficiency (Fig. 2A, B). These results would suggest an enhancement of ACC and/or ethylene synthesis in *Arabidopsis* roots under B deficiency. Accordingly, the external addition of ACC or ethephon (a compound that hydrolyses to ethylene above pH 3.5) to B-sufficient plants resulted in a clear reduction of LEH (Figs 3A, B, 4B).

It has been described that the short-term effect of ACC on root cell elongation is partially independent of its conversion to ethylene (Tsang *et al.*, 2011). In the present work, the inhibitory effect of ACC on LEH was mostly alleviated in the presence of silver ions (Ag^+^, an antagonist of ethylene perception) (Fig. 3A), which indicates that ACC exerts its action mainly after conversion to ethylene.

To investigate the possible mediation of ethylene in the effect of B deficiency on cell elongation, B deficiency was applied together with aminoethoxyvinylglycine (AVG, a chemical inhibitor of ACS) or Ag^+^. While AVG and Ag^+^ had no significant effect on LEH in the control treatment, both chemicals partially restored LEH in the B-deficient treatment (Fig. 4A). To further explore the possible role of ethylene on the inhibition of cell elongation under B deficiency, a genetic approach was performed by using the ethylene-insensitive *ein2-1* mutant. While the LEH of wild-type plants was strongly reduced by ACC or ethephon treatments, the LEH of *ein2-1* mutant was insensitive (Fig. 4B). In addition, LEH of the ethylene-insensitive mutant *ein2-1* was less sensitive to B deficiency than that of the wild-type plants (Fig. 4B).

Taken together, these results show that the rapid reduction of cell elongation triggered by B deficiency is mediated, in part, by an ethylene-dependent pathway. Therefore, we also wanted to study the possible contribution of the ethylene-independent pathway (via ACC per se) in the effect of B deficiency on cell elongation. Thus, the LEH response to B deficiency was tested in the presence of different Ag^+^ concentrations (see Supplementary Fig. S2 at *JXB* online). Interestingly, the inhibitory effect of B deficiency on LEH was not fully alleviated even in the presence of Ag^+^ concentrations as high as 100 or 200 μM (see Supplementary Fig. S2 at *JXB* online), which suggests that the involvement of ACC, as a signal in this response, cannot be completely ruled out.

### Involvement of auxin in the rapid inhibition of LEH under B deficiency

There is lots of evidence showing the synergistic interaction between ethylene and auxin in many different processes such as root growth inhibition and root hair formation (for a review, see Muday *et al.*, 2012). Therefore, experiments were conducted to analyse the possible involvement of auxin in the rapid inhibition of root cell elongation triggered by B deficiency.

**Fig. 5.**
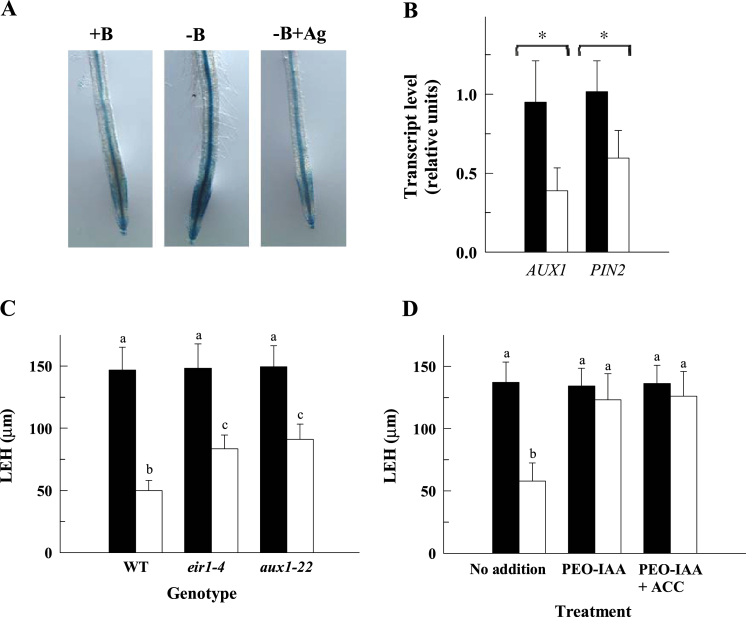
The inhibition of root cell elongation by B deficiency requires auxin signalling. (A) GUS expression in roots of IAA2::GUS seedlings grown in a B-deficient (0 μM B±10 μM Ag^+^) or control medium (10 μM B) for 4h. Images are representative individuals of two independent experiments with at least 10 seedlings examined for each experiment. (B) Quantitative RT-PCR analysis of *AUX1* and *PIN2* transcript levels in control (filled bars) and B-deficient (open bars) roots of wild-type plants after 4h of B treatment. The results are given as means ±SD (*n*=5 separate pools). Asterisks indicate statistically significant differences between B treatments according to Student’s *t* test (*P* <0.001). (C) The LEH parameter was measured in control (black bars) and B-deficient (white bars) roots of wild-type, *eir1-4*, and *aux1-22* mutant plants after 4h of B treatments. (D) The LEH parameter was measured in control (black bars) and B-deficient (white bars) roots after 4h of B treatment in the presence (or not) of 10 μM PEO-IAA or 10 μM PEO-IAA+10 μM ACC. The results are given as means ±SD (*n*=20 separate plants). Different letters are used to indicate means that differ significantly (*P* <0.001) according to Student’s *t* test. (This figure is available in colour at *JXB* online.)

The auxin reporter line IAA2::GUS (Swarup *et al.*, 2001) showed an increased expression in the stele throughout the root in response to B deficiency (Fig. 5A), which would indicate an increase in rootward auxin transport from the shoot. In addition, an accumulation of IAA2::GUS signals was also observed in the epidermis and cortex of the elongation and maturation zones of B-deficient roots (Fig. 5A), suggesting an enhancement in shootward auxin transport and signalling in these zones after 4h of B deficiency. To test this hypothesis, the expression levels of *AUX1* and *PIN2* genes, which are essential for shootward auxin transport between the root apical and elongation zone tissues (Růžička *et al.*, 2007), were analysed in B-sufficient and B-deficient roots (Fig. 5B). Curiously, B deficiency caused a decrease in the root expression of *AUX1* and *PIN2* genes (Fig. 5B). The response of LEH to B deficiency in *eir1-4* (*pin2*) and *aux1-22 Arabidopsis* was also studied. The LEH of both mutants was slightly less sensitive to B deficiency than that of wild-type plants (Fig. 5C), indicating the involvement of shootward auxin transport (via EIR1/PIN2 and AUX1) in the inhibition of root cell elongation under B deprivation. To further evaluate whether auxin modulates this inhibition, the response of LEH to contrasting B treatments in the presence of PEO-IAA, a synthetic antagonist of the TIR1 auxin receptor function (Hayashi *et al.*, 2008), was also studied. As shown, the effect of B deficiency on LEH was completely mitigated by PEO-IAA (Fig. 5D), which highlights the importance of auxin signalling in the inhibition of root cell elongation caused by B deficiency.

### Involvement of ROS production in the rapid inhibition of LEH under B deficiency

**Fig. 6.**
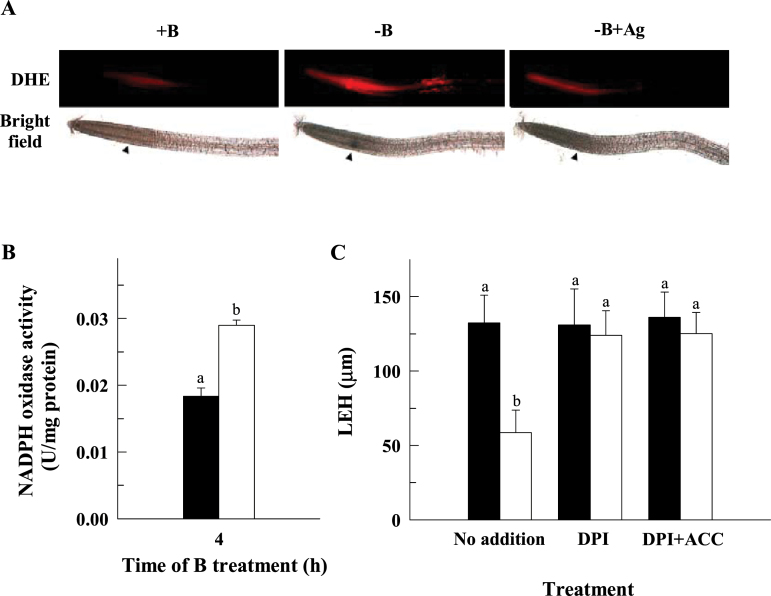
The inhibition of root cell elongation by B deficiency requires ROS signalling. (A) DHE staining in roots of *Arabidopsis* seedlings grown in a B-deficient (0 μM B±10 μM Ag^+^) or control medium (10 μM B) for 4h. Images are representative individuals of two independent experiments with at least 10 seedlings examined for each experiment. Arrowheads indicate the boundary between the meristematic and elongation zones of the root. (B) NADPH oxidase activity was measured in control (black bar) and B-deficient (white bar) roots after 4h of B treatment. The results are given as means ±SD (*n*=5 separate pools). (C) The LEH parameter was measured in control (black bars) and B-deficient (white bars) roots after 4h of B treatment in the presence (or not) of 10 μM DPI or 10 μM DPI+10 μM ACC. The results are given as means ±SD (*n*=20 separate plants). Different letters are used to indicate means that differ significantly (*P* <0.001) according to Student’s *t* test. (This figure is available in colour at *JXB* online.)

Previous studies have reported that B deficiency induces a rapid accumulation of ROS in tobacco BY-2 cells (Koshiba *et al.*, 2010) and in growing roots of *Arabidopsis* (Oiwa *et al.*, 2013). To ascertain whether the inhibition of LEH observed under B deficiency was also related to oxidative damage, *Arabidopsis* roots were stained with dihydroethidium (DHE), which enables the detection of ROS by fluorescence microscopy. Interestingly, B deficiency caused an increase of red fluorescence in the elongation zone of *Arabidopsis* roots (Fig. 6A), which suggests the possible involvement of ROS production in the inhibition of LEH under B deprivation. This hypothesis is also supported by the following two facts: (i) root NADPH oxidase activity, which is responsible for the generation of apoplastic ROS (Suzuki *et al.*, 2011), increased in response to B deficiency (Fig. 6B), and (ii) root cell elongation under B deficiency was alleviated in the presence of diphenylene iodonium (DPI, Fig. 6C), an inhibitor of ROS production mediated by flavoenzymes such as NADPH oxidases. Nevertheless, as DPI is not a specific inhibitor of NADPH oxidases, conclusions derived from the effects of DPI should be interpreted with caution and supported with other evidence.

### Auxin and ROS act downstream of ethylene in the rapid inhibition of LEH under B deficiency

Ethylene has been reported to act upstream of auxin and ROS in the response of *Arabidopsis* plants to several abiotic stresses (Jung *et al.*, 2009; Tsang *et al.*, 2011; Oiwa *et al.*, 2013). These facts have also been observed in the present work; thus the increased IAA2::GUS activity and DHE staining under B deficiency was abolished in the presence of Ag^+^ (Figs 5A, 6A). In addition, the effect of B deficiency on LEH was completely mitigated by PEO-IAA and DPI even in the presence of ACC (Figs 5D, 6C). Therefore, all these results would indicate that auxin and ROS act downstream of ethylene in the inhibition of LEH caused by B deficiency.

## Discussion

One of the early physiological effects observed when plants are subjected to B deficiency is the decrease of primary root growth (Fig. 1A; Dell and Huang, 1997; Martin-Rejano *et al.*, 2011), this effect being the result of a rapid inhibition in the elongation of root cells (Fig. 1C) rather than in cell division rate (Dell and Huang, 1997; Martin-Rejano *et al.*, 2011). During the rapid elongation phase of root cells its surface area increases notably, which requires massive cell wall rearrangement and *de novo* polysaccharide biosynthesis (Cosgrove, 1999). Therefore, the rapid inhibition of root cell elongation caused by B deficiency could be the consequence of the B requirement for cross-linking of the pectic polysaccharide rhamnogalacturonan II (RG-II) in cell walls (Kobayashi *et al.*, 1996). Thus, RG-II–B complex formation contributes to the maintenance of the mechanical properties of the cell wall structure (Ryden *et al.*, 2003; O’Neill *et al.*, 2004), which is decisive for the cell elongation process (De Cnodder *et al.*, 2005). Accordingly, it has been described that reduced cross-linking of RG-II by borate results in lower root growth in pumpkin (Matsunaga and Ishii, 2006) and *Arabidopsis* (Miwa *et al.*, 2013) plants. In addition, it has also been proposed that the expression of several genes coding for cell wall-modifying enzymes is down-regulated under B deficiency (Camacho-Cristóbal *et al.*, 2008a) which could alter the cell wall loosening required for cell elongation (Cosgrove, 1999).

Despite the above discussion, little is known about the underlying mechanisms that lead to an inhibition of cell elongation under B deficiency. Previously, evidence about the involvement of ethylene and auxin in the responses of root system architecture to low boron supply in *Arabidopsis* seedlings has been reported (Martín-Rejano *et al.*, 2011), which may suggest the occurrence of a signalling pathway controlling the response of root growth to B deficiency. To go deeply into this item, it was studied whether the rapid response of cell elongation to B deficiency could be altered by various compounds or in mutants related to these hormones. Regarding ethylene, both Ag^+^ (ethylene action blocker) and AVG (ACS inhibitor) mitigated the effect of B deficiency on cell elongation in wild-type plants (Fig. 4A); in addition, cell elongation of the ethylene-insensitive mutant *ein2-1* was less sensitive to B deficiency than that of wild-type plants (Fig. 4B). These observations suggest that the reduction of cell elongation triggered by B deficiency is not only a passive biomechanical consequence of weakened walls, but also an active process that depends on ethylene/ACC signalling. The up-regulation of the *ACS11* gene under B deficiency (Fig. 2) would also support this assumption.

It is well known that ethylene affects root growth by inhibiting the rapid expansion of cells leaving the root meristem (Le *et al.*, 2001; Swarup *et al.*, 2007). Tsang *et al.* (2011) have described an unconventional ACC-dependent mechanism that acts independently of ethylene signalling in the rapid inhibition of cell elongation triggered by cell wall stresses. These authors concluded that ACC per se has a short-term (few hours) influence on root cell elongation whereas, for long-term growth responses, the conversion of ACC to ethylene (ethylene-dependent pathway) is required (Tsang *et al.*, 2011). However, our results suggest a much larger contribution of the ethylene-dependent pathway in the control of the LEH response to 4-h B deficiency or ACC treatment when compared with the ACC-dependent one. This is supported by the following facts: (i) the inhibitory effect of B deficiency or ACC treatment on cell elongation was mostly alleviated in the presence of Ag^+^ (Figs 3A, 4A), and (ii) the LEH in the ethylene-insensitive *ein2-1* mutant was insensitive to ACC treatment (Fig. 4B). However, the results presented do not allow the involvement of ACC as a signal in the response of cell elongation to B deficiency to be completely ruled out, since Ag^+^ was not able to fully alleviate the inhibitory effect of B deficiency on cell elongation. Therefore, assuming that ACC acts in the short-term response of LEH to cell wall stress (Tsang *et al.*, 2011), our results would indicate that the ACC-dependent pathway is down-regulated in favour of the ethylene-dependent ones before 4h of B deficiency.

Cell elongation in roots is negatively controlled by ethylene which, in turn, requires auxin biosynthesis and transport (Růžička *et al.*, 2007; Stepanova *et al.*, 2007; Swarup *et al.*, 2007). The response of cell elongation to B deficiency follows this established pathway. Thus, the increased IAA2::GUS activity in the elongation zone (Fig. 5A) and the complete restoration of cell elongation by PEO-IAA (auxin signalling inhibitor) (Fig. 5D) in B-deficient roots indicate the requirement of auxin signalling in this response. Furthermore, the LEH response in *eir1-4* (*pin2*) and *aux1-22* mutants (Fig. 5C) suggests the partial involvement of shootward auxin transport via AUX1 and PIN2 in the inhibition of cell elongation under B deficiency. Therefore, our results seem to indicate that, despite the transcript levels of *AUX1* and *PIN*2 being reduced under B deficiency (Fig. 5B), sufficient auxin is still able to reach the elongation zone tissues to block cell elongation. Indeed, increased IAA2::GUS expression could simply result from increased cellular sensitivity to auxin upon B deficiency which still occurs despite lower levels of auxin reaching the elongation zone due to lower rates of shootward transport associated with lower levels of transporters. Hence, this would imply that B deficiency affects cell elongation and root growth through a process that involves altered auxin response in addition to polar auxin transport. A relationship between B deficiency and changes in auxin distribution has also been described in *Arabidopsis* root tips (Martín-Rejano *et al.*, 2011). Moreover, our results also show that auxin signalling acts downstream of ethylene in the response of cell elongation to B deficiency. In fact, Ag^+^ and PEO-IAA treatments were able to eliminate the effects of B deficiency on IAA2::GUS activity (Fig. 5A) and cell elongation even in the presence of ACC (Fig. 5D), respectively.

Besides ethylene and auxin, the involvement of ROS production in the rapid inhibition of root cell elongation under B deficiency is also indicated by our results. Thus, the increased DHE staining (Fig. 6A) and NADPH oxidase activity (Fig. 6B), and the capacity of DPI (ROS production inhibitor) to restore the length of the cells (Fig. 6C) in the elongation zone of *Arabidopsis* roots under B deficiency support this assumption. ROS production in roots is observed in response to a deficiency of several macronutrients and may be an important component in signalling nutrient deprivation (Schachtman and Shin, 2007). Previous studies have also suggested that B deficiency immediately leads to ROS accumulation in the root elongation zone of *Arabidopsis* plants which could cause oxidative damage and cell death in the same region (Oiwa *et al.*, 2013). ROS production has also been described to be involved in the inhibition of root cell elongation after ACC (De Cnodder *et al.*, 2005) or isoxaben (Tsang *et al.*, 2011) treatments. In agreement with these reports, ROS production acts downstream of ethylene in the pathway that results in the inhibition of root cell elongation (Fig. 6).

In summary, our results show that a signalling pathway involving ethylene, auxin, and ROS participates in the reduction of root cell elongation when *Arabidopsis* seedlings are subjected to B deficiency. This signalling pathway seems to be triggered by impaired cell wall integrity caused by B deficiency; in fact, a similar signalling process has been described to reduce root elongation rapidly under various types of cell wall stress (Tsang *et al.*, 2011). Nonetheless, further research is needed to elucidate the possible participation of other signalling molecules in the pathway that transmits the signal of B deficiency from the cell wall to the cytoplasm. Thus, for instance, several reports have described the involvement of calcium in the early response of tobacco BY-2 cells (Koshiba *et al.*, 2010) and *Arabidopsis* root (Quiles-Pando *et al.*, 2013; González-Fontes *et al.*, 2014) to B deprivation. Interestingly, it has recently been shown that the phosphorylation of RBOHD protein by a calcium-dependent protein kinase is required for activating ROS production (Dubiella *et al.*, 2013), suggesting that calcium is involved in the propagation of the ROS wave (Suzuki *et al.*, 2013). Furthermore, it has been proposed that the signalling network dependent on calcium and ROS may play a key role in regulating the mechanical properties of the cell wall, metabolic changes, and gene expression (Kurusu *et al.*, 2013). In addition to this, future research should also be directed to study the possible occurrence of a dedicated system to sensing the loss of cell wall integrity under B deficiency; in this way, different receptor-like kinase families have been considered as possible sensors for the perception of cell wall damage (for a review, see Engelsdorf and Hamann, 2014).

## Supplementary data

Supplementary data can be found at *JXB* online.

Supplementary Fig. S1. GUS expression in roots of proACS[2…9]::GUS seedlings grown in a B-deficient or a control medium over 4h.

Supplementary Fig. S2. Dose–response curve of LEH to external Ag^+^ concentrations in control and B-deficient roots after 4h of B treatment.

Supplementary Data

## References

[CIT0001] Ahmad W Zia MH Malhi SS Niaz A Saifullah 2012 Boron deficiency in soils and crops: a review. In: Goyal A, ed. Crop plant. InTech, 77–114. doi: 10.5772/36702

[CIT0002] Beato VM Rexach J Navarro-Gochicoa MT Camacho-Cristóbal JJ Herrera-Rodríguez MB Maldonado JM González-Fontes A 2010 A tobacco asparagine synthetase gene responds to carbon and nitrogen status and its root expression is affected under boron stress. Plant Science 178, 289–298.

[CIT0003] Bolaños L Lukaszewski K Bonilla I Blevins D 2004 Why boron? Plant Physiology and Biochemistry 42, 907–912. 15694285 10.1016/j.plaphy.2004.11.002

[CIT0004] Bradford MM 1976 A rapid and sensitive method for the quantitation of microgram quantities of protein utilizing the principle of the protein–dye binding. Analytical Biochemistry 72, 248–254. 942051 10.1016/0003-2697(76)90527-3

[CIT0005] Brown PH Bellaloui N Wimmer MA Bassil ES Ruiz J Hu H Pfeffer H Dannel F Römheld V 2002 Boron in plant biology. Plant Biology 4, 205–223.

[CIT0006] Cakmak I Römheld V 1997 Boron deficiency-induced impairments of cellular functions in plants. Plant and Soil 193, 71–83.

[CIT0007] Camacho-Cristóbal JJ Herrera-Rodríguez MB Beato VM Rexach J Navarro-Gochicoa MT Maldonado JM González-Fontes A 2008 *a* The expression of several cell wall-related genes in *Arabidopsis* roots is down-regulated under boron deficiency. Environmental and Experimental Botany 63, 351–358.

[CIT0008] Camacho-Cristóbal JJ Rexach J González-Fontes A 2008 *b* Boron in plants: deficiency and toxicity. Journal of Integrative Plant Biology 50, 1247–1255. 19017112 10.1111/j.1744-7909.2008.00742.x

[CIT0009] Camacho-Cristóbal JJ Rexach J Herrera-Rodríguez MB Navarro-Gochicoa MT González-Fontes A 2011 Boron deficiency and transcript level changes. Plant Science 181, 85–89. 21683871 10.1016/j.plantsci.2011.05.001

[CIT0010] Chormova D Messenger DJ Fry SC 2014 Boron bridging of rhamnogalacturonan-II, monitored by gel electrophoresis, occurs during polysaccharide synthesis and secretion but not post-secretion. The Plant Journal 77, 534–546. 24320597 10.1111/tpj.12403 PMC4171739

[CIT0011] Cosgrove DJ 1999 Enzymes and other agents that enhance cell wall extensibility. Annual Review of Plant Physiology and Plant Molecular Biology 50, 391–417. 10.1146/annurev.arplant.50.1.391 11541953

[CIT0012] De Cnodder T Vissenberg K Van Der Straeten D Verbelen JP 2005 Regulation of cell length in the *Arabidopsis thaliana* root by the ethylene precursor 1-aminocyclopropane-1-carboxylic acid: a matter of apoplastic reactions. New Phytologist 168, 541–550 16313637 10.1111/j.1469-8137.2005.01540.x

[CIT0013] Dell B Huang L 1997 Physiological response of plants to low boron. Plant and Soil 193, 103–120.

[CIT0014] Dubiella U Seybold H Durian G Komander E Lassig R Witte CP Schulze WX Romeis T 2013 Calcium-dependent protein kinase/NADPH oxidase activation circuit is required for rapid defense signal propagation. Proceedings of the National Academy of Sciences, USA 110, 8744–8749. 10.1073/pnas.1221294110 PMC3666735 23650383

[CIT0015] Engelsdorf T Hamann T 2014 An update on receptor-like kinase involvement in the maintenance of plant cell wall integrity. Annals of Botany 114, 1339–1347. 24723447 10.1093/aob/mcu043 PMC4195549

[CIT0016] Goldbach HE Wimmer M 2007 Boron in plants and animals: is there a role beyond cell-wall structure? Journal of Plant Nutrition and Soil Science 170, 39–48.

[CIT0017] González-Fontes A Rexach J Quiles-Pando C Herrera-Rodríguez MB Camacho-Cristóbal JJ Navarro-Gochicoa MT 2013 Transcription factors as potential participants in the signal transduction pathway of boron deficiency. Plant Signaling & Behavior 8, e26114. 23989264 10.4161/psb.26114 PMC4091350

[CIT0018] González-Fontes A Navarro-Gochicoa MT Camacho-Cristóbal JJ Herrera-Rodríguez MB Quiles-Pando C Rexach J 2014 Is Ca^2+^ involved in the signal transduction pathway of boron deficiency? New hypotheses for sensing boron deprivation. Plant Science 217–218, 135–139. 10.1016/j.plantsci.2013.12.011 24467905

[CIT0019] Hayashi K Hatate T Kepinski S Nozaki H 2008 Design and synthesis of auxin probes specific to TIR1, auxin receptor. Regulation of Plant Growth & Development, Supplement 43.

[CIT0020] Herrera-Rodríguez MB González-Fontes A Rexach J Camacho-Cristóbal JJ Maldonado JM Navarro-Gochicoa MT 2010 Role of boron in vascular plants and response mechanisms to boron stress. Plant Stress 4, 115–122.

[CIT0021] Jung JY Shin R Schachtman DP 2009 Ethylene mediates response and tolerance to potassium deprivation in *Arabidopsis* . The Plant Cell 21, 607–621. 19190240 10.1105/tpc.108.063099 PMC2660615

[CIT0022] Kobayashi M Matoh T Azuma J 1996 Two chains of rhamnogalacturonan II are cross-linked by borate-diol ester bonds in higher plant cell walls. Plant Physiology 110, 1017–1020. 12226238 10.1104/pp.110.3.1017 PMC157802

[CIT0023] Koshiba T Kobayashi M Ishihara A Matoh T 2010 Boron nutrition of cultured tobacco BY-2 cells. VI. Calcium is involved in early responses to boron deprivation. Plant & Cell Physiology 51, 323–327. 20008940 10.1093/pcp/pcp179

[CIT0024] Kurusu T Kuchitsu K Nakano M Nakayama Y Iida H 2013 Plant mechanosensing and Ca^2+^ transport. Trends in Plant Science 18, 227–233. 23291244 10.1016/j.tplants.2012.12.002

[CIT0025] Le J Vandenbussche F Van Der Straeten D Verbelen J-P 2001 In the early response of *Arabidopsis* roots to ethylene, cell elongation is up- and down regulated and uncoupled from differentiation. Plant Physiology 125, 519–522. 11161008 10.1104/pp.125.2.519 PMC1539361

[CIT0026] Martín-Rejano EM Camacho-Cristóbal JJ Herrera-Rodríguez MB Rexach J Navarro-Gochicoa MT González-Fontes A 2011 Auxin and ethylene are involved in the responses of root system architecture to low boron supply in *Arabidopsis* seedlings. Physiologia Plantarum 142, 170–178. 21338369 10.1111/j.1399-3054.2011.01459.x

[CIT0027] Matsunaga T Ishii T 2006 Borate cross-linked/total rhamnogalacturonan II ratio in cell walls for the biochemical diagnosis of boron deficiency in hydroponically grown pumpkin. Analytical Sciences 22, 1125–1127. 16896255 10.2116/analsci.22.1125

[CIT0028] Miwa K Wakuta S Takada S Ide K Takano J Naito S Omori H Matsunaga T Fujiwara T 2013 Roles of BOR2, a boron exporter, in cross-linking of rhamnogalacturonan II and root elongation under boron limitation in *Arabidopsis* . Plant Physiology 163, 1699–1709. 24114060 10.1104/pp.113.225995 PMC3850200

[CIT0029] Muday GK Rahman A Binder BM 2012 Auxin and ethylene: collaborators or competitors? Trends in Plant Science 17, 181–195. 22406007 10.1016/j.tplants.2012.02.001

[CIT0030] Oiwa Y Kitayama K Kobayashi M Matoh T 2013 Boron deprivation immediately causes cell death in growing roots of *Arabidopsis thaliana* (L.) Heynh. Soil Science & Plant Nutrition 59, 621–627.

[CIT0031] O’Neill MA Ishii T Albersheim P Darvill AG 2004 Rhamnogalacturonan II: structure and function of a borate cross-linked cell wall pectic polysaccharide. Annual Review of Plant Biology 55, 109–139. 10.1146/annurev.arplant.55.031903.141750 15377216

[CIT0032] Ozgur R Turkan I Uzilday B Sekmen AH 2014 Endoplasmic reticulum stress triggers ROS signalling, changes the redox state, and regulates the antioxidant defence of *Arabidopsis thaliana* . Journal of Experimental Botany 65, 1377–1390. 24558072 10.1093/jxb/eru034 PMC3969530

[CIT0033] Quiles-Pando C Rexach J Navarro-Gochicoa MT Camacho-Cristóbal JJ Herrera-Rodríguez MB González-Fontes A 2013 Boron deficiency increases the levels of cytosolic Ca^2+^ and expression of Ca^2+^-related genes in *Arabidopsis thaliana* roots. Plant Physiology and Biochemistry 65, 55–60. 23416496 10.1016/j.plaphy.2013.01.004

[CIT0034] Refrégier G Pelletier S Jaillard D Höfte H 2004 Interaction between wall deposition and cell elongation in dark-grown hypocotyl cells in *Arabidopsis* . Plant Physiology 135, 959–968. 15181211 10.1104/pp.104.038711 PMC514130

[CIT0035] Růžička K Ljung K Vanneste S Podhorská R Beeckman T Friml J Benková E 2007 Ethylene regulates root growth through effects on auxin biosynthesis and transport-dependent auxin distribution. The Plant Cell 19, 2197–2212. 17630274 10.1105/tpc.107.052126 PMC1955700

[CIT0036] Ryden P Sugimoto-Shirasu K Smith AC Findlay K Reiter WD McCann MC 2003 Tensile properties of *Arabidopsis* cell walls depend on both a xyloglucan cross-linked microfibrillar network and rhamnogalacturonan II-borate complexes. Plant Physiology 132, 1033–1040. 12805631 10.1104/pp.103.021873 PMC167041

[CIT0037] Schachtman D Shin R 2007 Nutrient sensing and signaling: NPKS. Annual Review of Plant Biology 58, 47–69. 10.1146/annurev.arplant.58.032806.103750 17067284

[CIT0038] Shorrocks VM 1997 The occurrence and correction of boron deficiency. Plant and Soil 193, 121–148.

[CIT0039] Stepanova AN Yun J Likhacheva AV Alonso JM 2007 Multilevel interactions between ethylene and auxin in *Arabidopsis* roots. The Plant Cell 19, 2169–2185. 17630276 10.1105/tpc.107.052068 PMC1955696

[CIT0040] Suzuki N Miller G Morales J Shulaev V Torres MA Mittler R 2011 Respiratory burst oxidases: the engines of ROS signaling. Current Opinion in Plant Biology 14, 691–699. 21862390 10.1016/j.pbi.2011.07.014

[CIT0041] Suzuki N Miller G Salazar C 2013 Temporal–spatial interaction between reactive oxygen species and abscisic acid regulates rapid systemic acclimation in plants. The Plant Cell 25, 3553–3569. 24038652 10.1105/tpc.113.114595 PMC3809549

[CIT0042] Swarup R Friml J Marchant A Ljung K Sandberg G Palme K Bennett M 2001 Localization of the auxin permease AUX1 suggests two functionally distinct hormone transport pathways operate in the Arabidopsis root apex. Genes & Development 15, 2648–2653. 11641271 10.1101/gad.210501 PMC312818

[CIT0043] Swarup R Perry P Hagenbeek D Van Der Straeten D Beemster GTS Sandberg G Bhalerao R Ljung K Bennett MJ 2007 Ethylene regulates auxin biosynthesis in *Arabidopsis* seedlings to enhance inhibition of root cell elongation. The Plant Cell 19, 2186–2196. 17630275 10.1105/tpc.107.052100 PMC1955695

[CIT0044] Takano J Miwa K Fujiwara T 2008 Boron transport mechanisms: collaboration of channels and transporters. Trends in Plant Science 13, 451–457. 18603465 10.1016/j.tplants.2008.05.007

[CIT0045] Tsang DL Edmond C Harrington JL Nüshse TS 2011 Cell wall integrity controls root elongation via a general 1-aminocyclopropane-1-carboxylic acid-dependent, ethylene-independent pathway. Plant Physiology 156, 596–604. 21508182 10.1104/pp.111.175372 PMC3177261

[CIT0046] Tsuchisaka A Theologis A 2004 Unique and overlapping expression patterns among the *Arabidopsis* 1-Amino-cyclopropane-1-carboxylate synthase gene family members. Plant Physiology 136, 2982–3000. 15466221 10.1104/pp.104.049999 PMC523360

