# Supplementary material for: Boron deficiency inhibits root cell elongation via an ethylene/auxin/ROS-dependent pathway in Arabidopsis seedlings
Source: J Exp Bot. 2015 Apr 28;66(13):3831–40. doi: 10.1093/jxb/erv186 (PMC4473985; doi:10.1093/jxb/erv186)
Supplement: Supplementary Data [file supp_66_13_3831__index.html]

Boron deficiency inhibits root cell elongation via an ethylene/auxin/ROS-dependent pathway in Arabidopsis seedlings — Boron deficiency inhibits root cell elongation via an ethylene/auxin/ROS-dependent pathway in Arabidopsis seedlings — Supplementary Data 

# Boron deficiency inhibits root cell elongation via an ethylene/auxin/ROS-dependent pathway in *Arabidopsis* seedlings

## Supplementary Data

Data files

**Files in this Data Supplement:**

- Supplementary Data - Supplementary Data
