# Supplementary material for: Boron deficiency inhibits root cell elongation via an ethylene/auxin/ROS-dependent pathway in Arabidopsis seedlings
Source: J Exp Bot. 2015 Apr 28;66(13):3831–40. doi: 10.1093/jxb/erv186 (PMC4473985; doi:10.1093/jxb/erv186)
Supplement: Supplementary Data [file supp_erv186_jexbot144402_file001.pdf]

Supplementary Data

Title: Boron deficiency inhibits root cell elongation via an ethylene/auxin/ROS dependent pathway in Arabidopsis seedlings

Authors: Juan J. Camacho-Cristóbal, Esperanza M. Martín-Rejano, M. Begoña Herrera-Rodríguez, M. Teresa Navarro-Gochicoa, Jesús Rexach, and Agustín González-Fontes

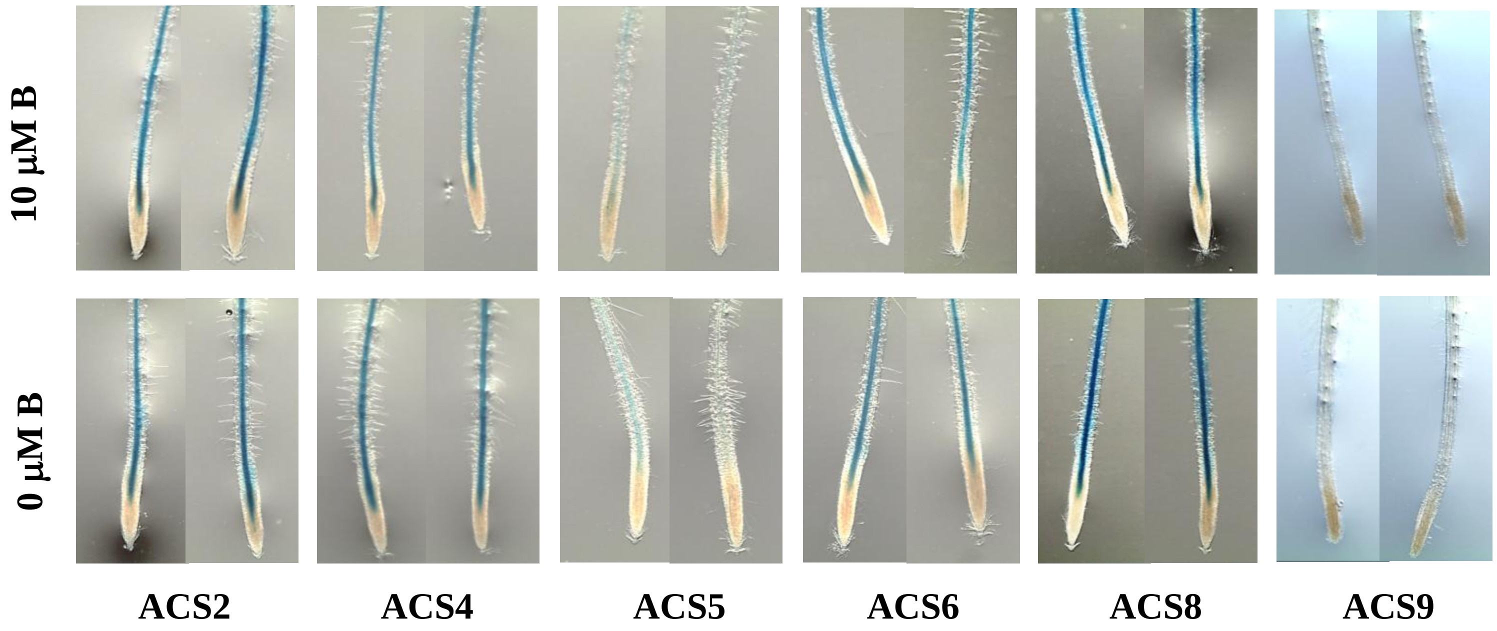

Figure S1. GUS expression in roots of proACS[2…9]::GUS seedlings grown in a B deficient (0 µM B) or control (10 µM B) medium during 4 h. Images are representative individuals of two independent experiments with at least 10 seedlings examined for each experiment.

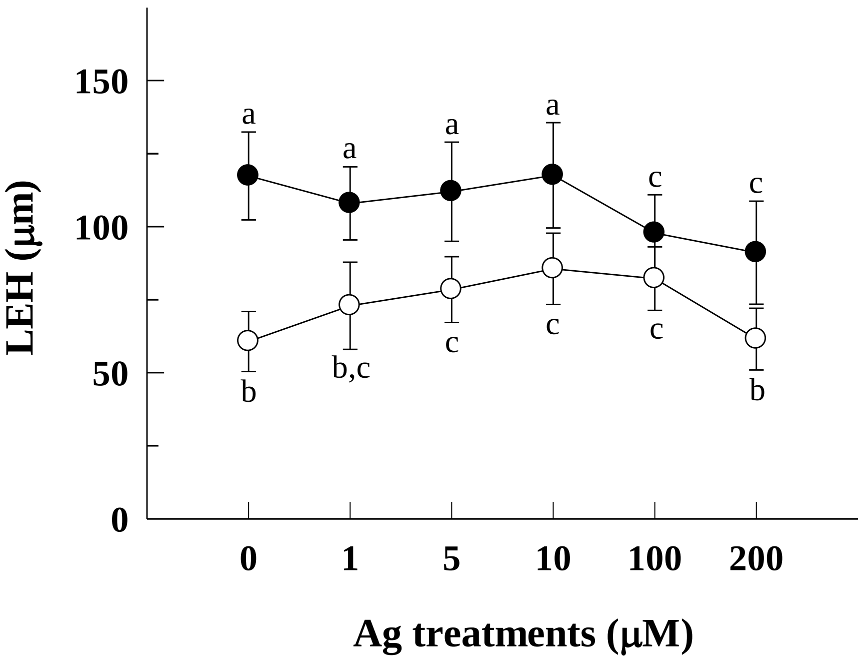

Figure S2. Dose-response curve of LEH to external Ag^{+} concentrations in control (filled circle) and B-deficient (open circle) roots after 4 h of B treatments. The results are given as means ± SD (n = 12 separate plants). Different letters are used to indicate means that differ significantly (P < 0.001) according to Student's t-test.
